# Novel fluorescent genome editing reporters for monitoring DNA repair pathway utilization at endonuclease-induced breaks

**DOI:** 10.1093/nar/gkt872

**Published:** 2013-10-09

**Authors:** Ryan Kuhar, Kamila S. Gwiazda, Olivier Humbert, Tyler Mandt, Joey Pangallo, Michelle Brault, Iram Khan, Nancy Maizels, David J. Rawlings, Andrew M. Scharenberg, Michael T. Certo

**Affiliations:** ^1^Center of Immunity and Immunotherapies, Seattle Children’s Research Institute, Seattle, 98101 Washington, USA, ^2^Program in Molecular and Cellular Biology, University of Washington, Seattle, 98195 Washington, USA, ^3^Department of Immunology, University of Washington, Seattle, 98195 Washington, USA and ^4^Department of Biochemistry, University of Washington, Seattle, 98195 Washington, USA

## Abstract

The creation of a DNA break at a specific locus by a designer endonuclease can be harnessed to edit a genome. However, DNA breaks may engage one of several competing repair pathways that lead to distinct types of genomic alterations. Therefore, understanding the contribution of different repair pathways following the introduction of a targeted DNA break is essential to further advance the safety and efficiency of nuclease-induced genome modification. To gain insight into the role of different DNA repair pathways in resolving nuclease-induced DNA breaks into genome editing outcomes, we previously developed a fluorescent-based reporter system, designated the Traffic Light Reporter, which provides a readout of gene targeting and gene disruption downstream of a targeted DNA double-strand break. Here we describe two related but novel reporters that extend this technology: one that allows monitoring of the transcriptional activity at the reporter locus, and thus can be applied to interrogate break resolution at active and repressed loci; and a second that reads out single-strand annealing in addition to gene targeting and gene disruption. Application of these reporters to assess repair pathway usage in several common gene editing contexts confirms the importance that chromatin status and initiation of end resection have on the resolution of nuclease-induced breaks.

## INTRODUCTION

Endonuclease-mediated genome editing involves the introduction of a targeted DNA double-strand break (DSB) in a live cell by a designer endonuclease, followed by resolution of the break by endogenous cellular DNA repair pathways that result in altered genomic information (1–3). As there are now multiple platforms available for creating site-specific endonucleases, including zinc-finger nucleases (ZFNs) (4,5), LAGLIDADG homing endonucleases (LHEs) (6,7), transcription activator-like effector nucleases (TALENs) (8,9) and RNA-guided endonucleases (RGENs) (10–12), effort can be focused on understanding how chromatin affects target accessibility and break-repair for different endonuclease platforms, and on developing strategies to control the resolution of breaks in order to efficiently and precisely attain the desired editing outcome (13,14).

DSB repair can proceed by one of several mechanisms, each of which can result in distinct genome editing outcomes. In classic nonhomologous end-joining (cNHEJ), break ends are rapidly recognized by Ku proteins together with DNA-dependent protein kinase (15,16), limiting end resection and the DNA is subsequently rejoined either seamlessly or with minimal processing that can result in small deletions and insertions. This pathway can be harnessed by designer endonucleases to disrupt the coding sequence of a gene to generate a knockout (gene disruption, GD) (17). Conversely, breaks can be recognized by the MRN complex, leading to the recruitment of specialized exonucleases and extensive single-strand 5′-end resection (18,19). The 3′ single-stranded DNA tails that remain may be resolved by ‘alternative-end joining’, in which microhomologies drive joining of proximal ends of the break with the generation of relatively small deletions (a pathway which can also be harnessed for targeted GD) (20,21); single-strand annealing (SSA), where resection between stretches of homologous DNA on either side of the break results in deletion of the intervening DNA; and homologous recombination, where the 3′-ends locate a fragment of homologous DNA, driving recombination between the region surrounding the DSB and the external sequence. The latter pathway can be harnessed by designer endonucleases to drive gene targeting (GT) with an exogenously provided template DNA, yielding precise genetic modifications such as reversion, introduction of point mutations or transmitting larger swaths of DNA at particular locations. Importantly, the range of possible genomic alterations downstream of a DNA break has been proposed to be determined by a stochastic ‘competition’ among the various repair pathways, the results of which may be influenced by a number of factors including cell cycle (22), the etiology of the break (23) and local chromatin structure (24).

Fluorescent reporter systems have proven indispensable for enabling rapid and sensitive evaluation of the different DNA repair pathways (3,25,26). To extend these systems to evaluate alterations in DNA repair outcomes produced by pharmacological or molecular and cell biological manipulations in the context of genome editing, we previously developed the Traffic Light Reporter (TLR) (27). This reporter allows for the simultaneous fluorescent measurement of GT and GD following expression of a site-specific endonuclease. Using this system, we have been able to evaluate and develop a number of manipulations that bias genome editing outcome downstream of the breakpoint, including identification of siRNAs that increase GT (27), application of single-strand breaks to allow GT while minimizing the incidence of mutagenic NHEJ (27,28), and coupling endonucleases to DNA end-processing enzymes to drive high rates of GD (13).

To better understand how utilization of each potential DNA repair pathway is influenced by variables such as the nuclease platform, chromatin context or by manipulations designed to bias break resolution toward a particular pathway, we have developed two novel TLR-based variants. The first provides continual readout of transcriptional activity at the TLR locus (Active/Repressed TLR, ‘AR-TLR’), which we have applied to assess the resolution of breaks located in loci that are active versus silent. The second adds a concurrent fluorescent repair readout of SSA to the original TLR (‘SSA-TLR’), providing information regarding the prevalence of 5′-end resection and its influence on genome editing outcome. This reporter system can be applied to learn how to harness SSA-like mechanisms for GT or GD, or to understand the consequence of editing within or near repetitive genomic regions. Together with the TLR, these reporter systems provide a suite of tools for studying how intrinsic cellular DNA repair pathways respond to endonuclease-induced DNA breaks, and for understanding how these pathways can be manipulated to generate more precise and efficient genome editing results in various genomic contexts.

## MATERIALS AND METHODS

### Construct assembly

Nuclease expression constructs used in all assays except for the SSA-TLR donor titration and nickase experiments were cloned into the mammalian expression plasmid ‘pExodus’ using standard molecular biology techniques. The truncated GFP donor template necessary for GT experiments was cloned in upstream of the nuclease portion of the expression constructs so nuclease expression and donor delivery could be monitored together. The near-infrared fluorescent protein (iRFP) sequence was obtained from Addgene (Accession number 31857). Nuclease expression constructs used for donor titration experiments in the SSA-TLR were cloned into the mammalian pCVL and pRRL lentiviral backbone. See Supplementary Figure S1 for plasmid maps. The AR-TLR and SSA-TLR reporters are available through the Addgene DNA repository.

### Cell-line derivation

AR-TLR cell lines were created by transducing 0.2 × 10^6^ HEK293T cells with limiting dilutions of reporter lentivirus. Three days posttransduction, the culture exhibiting <5% iRFP fluorescent cells were sorted using a BD FACSAriaII to isolate a heterogeneous population of highly iRFP fluorescent cells. Following this initial sort, cells were cultured for 2 weeks before undergoing a second sort to isolate a heterogeneous population of cells that had lost their iRFP fluorescence due to cell-intrinsic silencing of the lentiviral SFFV promoter.

The SSA-TLR cell lines were created by linearizing 1 µg of the reporter plasmid with ScaI, and then electroporating 1 × 10^7^ HEK293T cells at 250 V, 950 µF and infinite resistance using a Genepulser XcelTM Electroporator (BioRad). Cells were then plated in 15 cm dishes for 72 h to recover, followed by limiting dilution in puromycin-containing media to isolate a clonal population. Several clones for each reporter cell line were selected and analyzed on a BD LSRII for mCherry and iRFP fluorescence. The clone with the lowest background fluorescence was selected as the cell line to be used for experiments.

### Transfections, flow cytometry and analysis

For each experiment 1 × 10^5^ reporter-containing HEK293T cells were seeded in a 24-well plate 24 h prior to transfection. Cells were then transiently transfected with 0.5 µg of nuclease expression construct using X-tremeGENE9 DNA transfection reagent (Roche Applied Science) according to manufacturer protocol. Twenty-four hours after transfection, half the cells from each well were removed and fresh media was added. Cells were harvested 72 h posttransfection/transduction and analyzed on a BD LSRII for mTagBFP (405 nm laser for excitation, 450/50 filter for detection), mCherry (561 nm laser for excitation, 610/20 filter for detection), GFP (488 nm laser for excitation, 530/30 filter for detection) and iRFP (640 nm laser for detection, 710/50 filter for detection) fluorescence using an appropriate compensation matrix. Data were analyzed using FloJo software (TreeStar, Inc.). For sorting experiments, cells were sorted on BD Facs ARIAII.

### Evaluation of I-Sce I-induced mutagenesis by restriction digest

iRFP positive and negative AR-TLR cell lines were transfected with I-Sce I according to the protocol described above. Seventy-two hours after transfection 2.5 × 10^5^ BFP positive cells (to normalize for transfection efficiency) were isolated using a BD FACSAriaII and genomic DNA from the cells was extracted using a DNeasy® Blood & Tissue Kit (Qiagen). An amount of 100 ng DNA was then used for touchdown PCR using primers (Forward CACGATGTCGATCTCGATTTT Reverse GGGTGTTCTGCTGGTAGTGG) that amplify a 776-bp fragment with the embedded Sce site in the middle. Five-hundred nanograms of each PCR product was then digested with recombinant I-Sce I (NEB) for 1 h at 37°C before being run on an agarose gel and photographed. Band intensity was quantified using ImageJ software (NIH). To calculate overall percent modification of the locus we used the following formula: Resistant Band Intensity/(Resistant Band Intensity + Cleaved Band Intensity). For calculation of fold loss in efficiency of GD we divided the percent modification observed in the iRFP positive cell line by that observed in the iRFP negative.

### Restoration of fluorescent readout in iRFP negative AR-TLR cells

To reactivate the AR-TLR iRFP negative population, 2 × 10^5^ cells were grown in a 12-well plate for 96 h in 10 nM 5-Aza-2′-Deoxycitidine (5-aza-dC; Sigma). Due to the short half-life of 5-Aza, fresh drug was added to the culture daily. Analysis of iRFP fluorescence was performed daily on a BD LSRII.

### Lentivirus generation and transduction

Lentivirus for AR-TLR cell line derivation and SSA-TLR Donor response curve was produced by transient cotransfection of HEK293T cells in 10-cm dishes in 10 ml of medium using PEI transfection reagent (Polysciences) with 6 μg RRL or CVL backbone plamids, 1.5 μg pMD2G envelope plasmid (VSV-G). An amount of 3 μg psPAX2 was used for integrating lentivirus and psPAX2 D64V was used for integration-deficient lentivirus, per plate. Integrating lentivirus used for cell-line derivation or I-Sce I delivery was harvested from 293 T supernatant and stored at −80°C. Integrase deficient lentivirus was isolated by harvesting supernatant and concentrating 100× by overnight centrifugation at 8000*g*. The 100× stocks were aliquoted and stored at −80°C. Virus was quantified using Lenti-x p24 rapid titer ELISA kit (Clontech) according to the manufacturer's protocol. For transduction, 0.1 × 10^6^ HEK293T cells were seeded in a 24-well plate. The next day, cells were transduced with the amounts of lentivirus or integrase-deficient lentivirus as indicated. Transductions were done in the presence of 4 μg of polybrene. Twenty-four hours after transduction, medium was changed and cells were passaged to six-well plates and analyzed 48 h later (72 h posttransduction).

### Bisuflite sequencing

An amount of 1 µg genomic DNA was treated with sodium bisulfite to convert C to U, using the Epitect Bisulfite Kit (Qiagen) as recommended by the manufacturer. Primers for amplification of the reverse strand of the AR-TLR region from bisulfite-treated DNA were designed to lack CpG dinucleotides using Epidesigner (Sequenom). Primer sequences for nested amplification of the SFFV promoter were: F1, 5′-TAGGTTAAGAGGTTAGGTTGTTTGG; R1, 5′-CCAAACCAAAAATAAAAAAATTCAA; F2, 5′-TTGGAAATATTTGATGGGTTTTAAG. First round amplification was with F1/R1 and second round amplification was with F2/R1. Primers for nested amplification of the region surrounding the I-Sce I target site were: F3, 5′-TTTTAGTTTGTGTTTTAGGATGTTG; R3, 5′-AACCCTAAAATTCATCTACACCACC; F4, 5′-GTTGTGGTTGTTGTAGTTGTATTTT; R5, 5′-AAACCTACACTATCCTACCTCAACC. First round amplification was with F4/R5 and second round amplification was with F3/R3. DNA was amplified with Taq polymerase (NEB) and PCR fragments were purified from gels using the DNA extraction kit (Qiagen) and cloned into pCR2.1-TOPO TA vector (Invitrogen). Inserts were amplified with primers M13R/F (Invitrogen), purified and sequenced (Eurofins MWG operon). Sequences were analyzed with QUMA software (Kumaki Y, NAR, 2008)

### SSA TLR PCR

I-Sce I SSA-TLR cells were transfected with BFP-tagged I-Sce I according to the protocol described earlier. At 72 h 2.5 × 10^5^ BFP+/iRFP+ and BFP-/iRFP− (negative control) cells were isolated using a BD FACSAriaII and genomic DNA was isolated using a DNeasy Blood & Tissue Kit (Qiagen). One hundred nanaograms genomic DNA from each sample was then used in PCR using Accuprime Pfx DNA Polymerase (Invitrogen). The following primer set was used for amplicon generation: Forward, AGCTGCAGTAACGCCATTTT Reverse, CACGGCGACTACTGCACTTA.

## RESULTS

### AR-TLR: fluorescent reporter for readout of GT and GD at transcriptionally active and repressed loci

**Figure 1.**
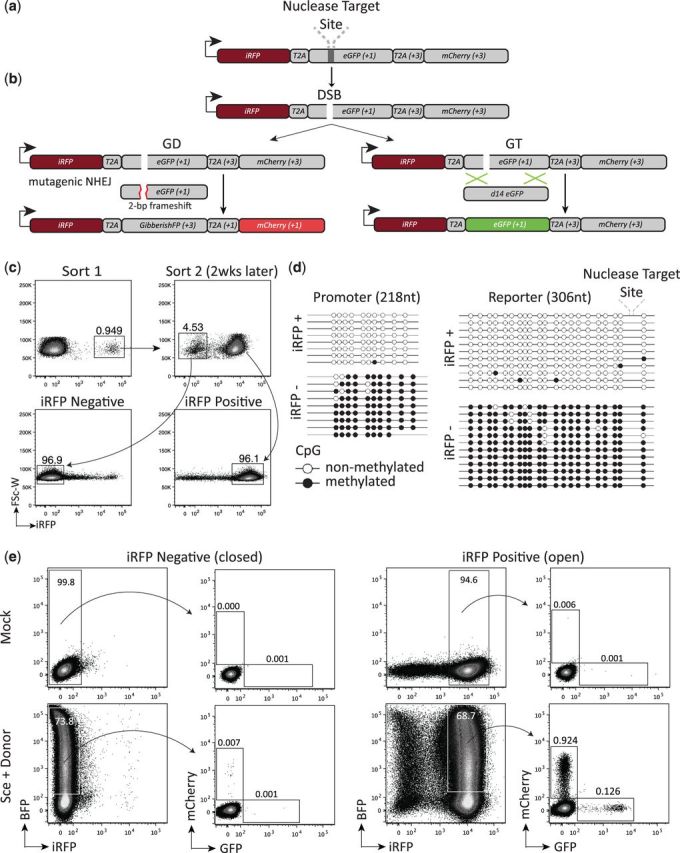
The AR-TLR. (**a**) Diagram of the AR-TLR. Arrow represents promoter and initial *iRFP* start codon. Reading frames relative to the initial *iRFP* start codon are indicated in parentheses. (**b**) Schematic showing the different genome engineering outcomes following the introduction of a DSB. If the break undergoes homologous GT the *eGFP* sequence is restored and the cell will fluoresce green. If the break undergoes gene disruption (GD/mutagenic NHEJ) resulting in a frameshift to the +3 reading frame, *eGFP* will be translated out of frame and the T2A and *mCherry* sequences will be translated in frame causing the cells to fluoresce red. (**c**) Representative flow plots depicting the flow cytometric-based method for deriving *iRFP+/−* populations of AR-TLR. (**d**) Depiction of bisulfite sequencing results generated from the two PCR amplicons of genomic DNA. Each circle corresponds to a CpG motif with a blank circle denoting nonmethylated CpG and black circle denoting methylated CpG. Sequences collected from the promoter region are shown on the left and those collected from the downstream reporter are shown on the right. (**e**) Flow cytometric analysis of HEK293T AR-TLR cells 72 h after transfection with the indicated pExodus constructs. Numbers shown inside plots indicate percentage of live cells. *BFP* expression is a marker for transfection efficiency.

To assess the contribution of chromatin structure on endonuclease-induced genome editing, we modified the previously characterized TLR by cloning a near-infrared fluorescent protein (29) coding sequence and T2A ‘de-linker’ immediately downstream of the spleen focus-forming virus (SFFV) promoter used to drive expression of the reporter (Figure 1a and b). Using iRFP fluorescence as a marker for transcriptional status of the reporter locus, we derived two distinct populations of cells to represent loci that are either open (transcriptionally active and iRFP+) or closed (transcriptionally inactive and iRFP−). This was accomplished by transducing a population of HEK293T cells with reporter-containing lentivirus at a low multiplicity of infection, and isolating a heterogeneous population of iRFP+ cells 3 days later using a flow sorter. The iRFP+ population was subsequently passaged for 2 weeks, allowing ∼5% of cells in which the reporter was initially transcriptionally active to become silenced by cell-intrinsic mechanisms, as indicated by the loss of iRFP fluorescence. This iRFP− population was then isolated on a flow sorter and expanded (Figure 1c).

To confirm that the iRFP− cells still contained reporter after losing their fluorescence and to assess the nature of the silencing, we isolated genomic DNA from both iRFP+ and iRFP− cells and analyzed their CpG methylation status through bisulfite sequencing (Figure 1d). A 218-bp amplicon located in the SFFV promoter revealed nearly complete methylation of all CpG motifs in the iRFP− population, whereas the iRFP+ population contained none. A 306-bp amplicon spanning the I-Sce I site gave similar results, namely near complete methylation of CpG motifs in the iRFP− population and absence of methylation in the iRFP+ cells. Overall, these results show that the AR-TLR reporter enables the isolation of cells that contain transcriptionally silent reporter due to CpG methylation from cells that contain an active and accessible reporter, allowing comparison of DNA repair profiles for each chromatin context.

### Influence of reporter silencing on genome editing outcome

As an initial assessment of how CpG methylation would effect repair of the reporter locus, we expressed BFP-tagged I-Sce I endonuclease with and without donor template in the iRFP+ and iRFP− populations and analyzed cells by flow cytometry (Figure 1e). The repair profile of the iRFP+ population was consistent with previous TLR work, depicting a bias toward GD (mCherry+) over GT (GFP+). The iRFP− population exhibited no fluorescent readout of repair despite the high level of nuclease expression, suggesting that the silenced loci are either inaccessible to the endonuclease, or that breaks are resolved with maintenance of the transcriptional status and fail to express the fluorescent repair readout.

**Figure 2.**
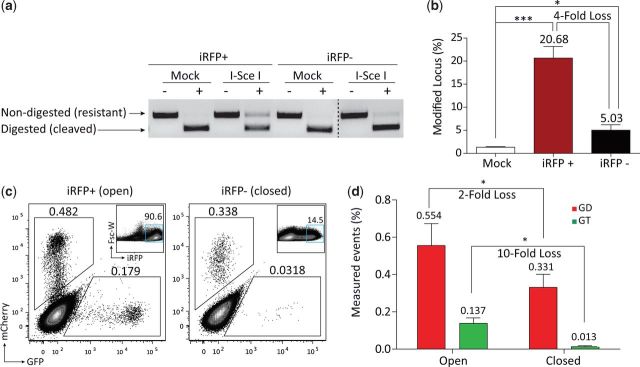
Assessing DNA repair pathway choice at open and closed loci. (**a**) Agarose gel showing results from digest of genomic DNA-generated amplicons with recombinant I-Sce I (denoted ‘+’ for containing recombinant I-Sce I and ‘−’ for no recombinant I-Sce I negative control). Nondigested (resistant) product is 776 bp and digested (cleaved) product is 388 bp. (**b**) Bar graph showing mean values for the amount of resistant (mutagenized) band observed over three replicates and fold loss in mutagenic product between *iRFP+/*− populations. *P < 0.05, ***P < 0.0005. *P*-values are shown in reference to mock. SEM is shown. (**c**) Flow cytometric analysis of HEK293T 5-aza-dC reactivated AR-TLR cells 2 weeks posttransfection with Sce + Donor pExodus expression constructs. Numbers shown adjacent to gates indicate percentage. Inset plots show locus reactivation and gating on *iRFP+* cells that were used for analysis. (**d**) Bar graph quantifying results from panel ‘c’. Values represent the mean from three independent experiments performed in duplicate with indicated *P*-values and fold loss.

To distinguish between these two possibilities, we first assessed whether I-Sce I was capable of producing any measurable cleavage-related events in the iRFP− cells. To accomplish this, we isolated and PCR-amplified the genomic DNA of nuclease-expressing (BFP+) iRFP+ and iRFP− cells and digested the products with recombinant I-Sce I (Figure 2a). In this assay, PCR products amplified from mutagenized target sites are resistant to digestion with recombinant I-Sce I, indicating that DNA breaks had occurred. Densitometric analysis of digest-resistant bands revealed a 4-fold loss of GD at the silenced loci (iRFP− = 5.03% ± 2.55 SEM) compared with their active counterparts (iRFP+ = 20.68% ± 1.18 SEM) (Figure 2b). These results indicate that I-Sce I was capable of accessing and cleaving the target site in the transcriptionally silenced cells, but at a lower efficiency as compared with transcriptionally active cells.

To determine if the loss of GD events at silenced loci was the result of reduced accessibility of the target site, or a bias in repair pathway choice, we sought to recover the fluorescent readout of repair in the iRFP− population so that we could compare the ratio of GD to GT between open and closed loci. To accomplish this, we treated the iRFP− cells for 96 h with an inhibitor of DNA methyltransferase activity, 5-aza-2′-deoxycitidine (5-aza-dC) (30) and observed ∼30% of the cells regain iRFP fluorescence (Supplementary Figure S2a). We confirmed that 5-aza-reactivated cells lost CpG methylation through bisulfite sequencing (Supplementary Figure S2b). To compare the repair pathway choice between the open and closed loci, we transfected both iRFP+/− populations with a single plasmid encoding I-Sce I nuclease and donor template tagged with a BFP tracking-fluorophore. Seventy-two hours posttransfection, nuclease-expressing cells were sorted from the population based on BFP expression (Supplementary Figure S3), and subsequently passaged until all nuclease expression had subsided (10 days postinitial transfection) (see Supplementary Figure S4 for schematic). The reporter locus was then reactivated by the addition of 5-aza, and GT and GD editing outcomes were compared between open and closed loci in cells expressing iRFP at similar fluorescent intensities (Figure 2c). We observed a mean 0.554% mCherry/0.137% GFP fluorescent cells in the open loci cells (originally iRFP+ population) and 0.331% mCherry/0.013% GFP fluorescence cells in the closed loci cells (originally iRFP− population) (Figure 2d). These results indicate that the repressive chromatin structure differentially affected the repair pathways, as GD events exhibited a 2-fold loss, whereas GT exhibited a relatively more substantial 10-fold loss. We also observed roughly half of the number of total repair events in the reactivated iRFP− population, likely a consequence of reduced accessibility of the target site to the endonuclease and/or repair factors. Finally, these results indicate that CpG methylation-induced transcriptional silencing of the locus has a more pronounced effect on usage of the GT pathway.

### SSA-TLR: fluorescent reporter for high-throughput analysis of SSA, GD and GT from the same breakpoint

**Figure 3.**
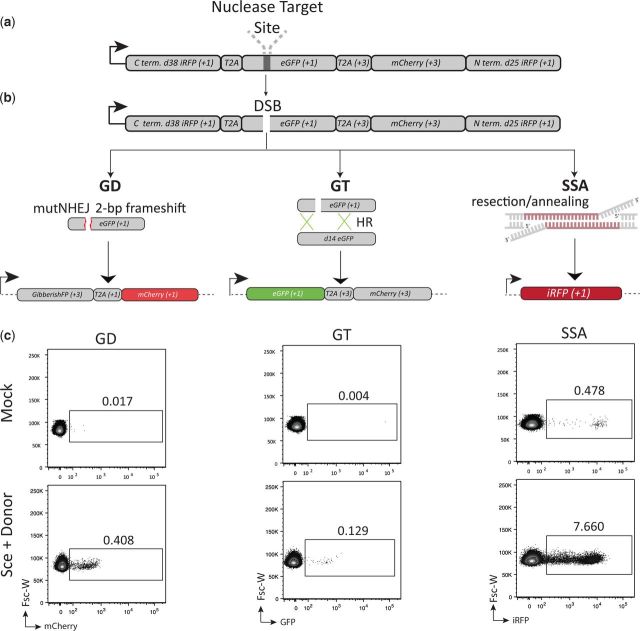
The SSA-TLR. (**a**) Diagram of the SSA-TLR. Arrow represents promoter and initial *iRFP* start codon. Reading frames relative to the initial *iRFP* start codon are indicated in parentheses. (**b**) Schematic showing the different genome editing outcomes after a DSB is made. The previously depicted GT and GD pathways remain the same as above and result in GFP and mCherry expression respectively. If the break undergoes repair via SSA, single-strand resection will reveal the homology between the two arms, which will subsequently be processed to result in a fully functional *iRFP* ORF. (**c**) Flow cytometric analysis of HEK293T SSA-TLR cells 72 h after transfection with the indicated pExodus constructs.

In order to simultaneously monitor utilization of SSA, GD and GT following a targeted DNA break, we modified the previously characterized TLR by flanking it with truncated iRFP arms (Figure 3a and b). Before a DSB is created, neither iRFP arm codes for a fully functional protein, as 38 amino acids were deleted from the C-terminus of the 5′ arm, and 25 amino acids from the N-terminus of the 3′-arm. However, if a DSB leads to sufficient resection to reveal the 762-bp homology between the two arms, it becomes possible for them to anneal and be processed into a complete iRFP coding sequence.

As an initial evaluation of reporter performance, we transiently transfected HEK293T cells from a single-cell clone harboring the SSA-TLR with I-Sce I nuclease and donor (Figure 3c). Surprisingly, I-SceI-induced breaks were highly prone to repair via the SSA pathway (iRFP+ = 6.42% ± 0.70 SEM), while GD and GT occurred at much lower rates (mCherry+ = 0.50 ± 0.05 SEM, GFP+ = 0.11 ± 0.04 SEM). We confirmed that iRFP+ events represented SSA-reconstituted full-length iRFP ORFs by using PCR to detect the loss of the intervening TLR sequence (Supplementary Figure S5). Importantly, the dominance of the SSA pathway was consistently seen in several distinct single-cell clones (Supplementary Figure S6), ruling out the possibility of a locus or cell specific effect. These findings suggest that fairly extensive 5′-end resection is a common occurrence following an I-SceI-induced DSB in HEK293T cells, and as a consequence, breaks with proximal flanking regions of homology may be extremely likely to resolve via SSA.

### Influence of DNA nicks and donor availability on repair pathway bias

**Figure 4.**
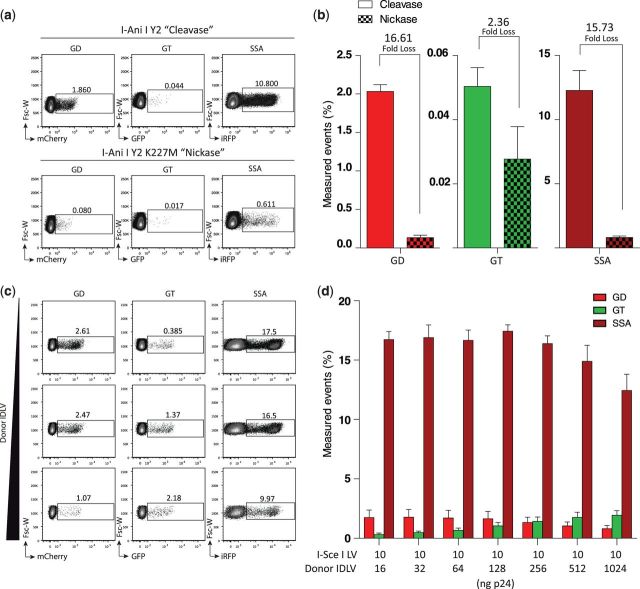
Influence of DNA nicks and donor availability on repair pathway bias. (**a**) Flow cytometric analysis of HEK293T SSA-TLR with I-Ani I target site 72 h after transfection with indicated nuclease + donor constructs. (**b**) Graph representing mean values from panel ‘a’ with fold loss (FL) between cleavase and nickase shown. (**c**) Flow cytometric analysis of HEK293T SSA-TLR cells with I-Sce I target site 72 h after transduction with indicated amounts of I-Sce I (LV) and donor template (IDLV). p24 values indicate the amount of lentiviral capsid proteins added to the cells. (**d**) Quantification of data from panel ‘c’. Bars represent three individually performed experiments with SEM shown.

DNA-nicking enzymes, ‘nickases’, have been reported to promote repair via the GT pathway while limiting mutagenic NHEJ (27,28,31). To assess the effect of DNA nicks on repair pathway bias between SSA, GD and GT, we used an SSA-TLR HEK293T cell line containing a target site for the LAGLIDADG homing endonuclease I-Ani I. We transfected the cell line with the engineered I-Ani I Y2 cleavase (32) or the I-Ani I Y2 K227M nickase (33) variant along with donor template (Figure 4a). When we compared the event rates of the cleavase with the nickase we observed a 16-fold loss in both GD and SSA; however, we observed only a 2.4-fold loss in GT (Figure 4b). These findings agree with previous work showing that DNA nicks reduce mutagenic NHEJ relative to GT, and, suggest that DNA nicks also appear to limit the prevalence of SSA compared with GT.

Our previous work with the TLR evaluated the effect of increasing copies of donor template on repair pathway bias between GD and GT, finding a dose-dependent relationship between donor availability and GT event rate (27). Therefore, we reasoned that increasing donor availability might increase the competitiveness of the GT pathway and attenuate SSA dominance. To accomplish this we used an integration-deficient lentivirus (IDLV) to provide increasing amounts of donor template to the cell while holding the amount of expressed I-Sce I constant. In these experiments, we found that in addition to the previously observed donor-dependent inverse relationship between GD and HGT (27), increasing amount of donor template also effectively competes for events that would otherwise be resolved by SSA pathways (Figure 4c and d). This finding is consistent with the idea that altNHEJ, GT and SSA all proceed following end resection, and that locating exogenously provided donor templates during a homology search is limiting for GT outcomes.

### Effect of endonuclease platform on break repair pathway choice

**Figure 5.**
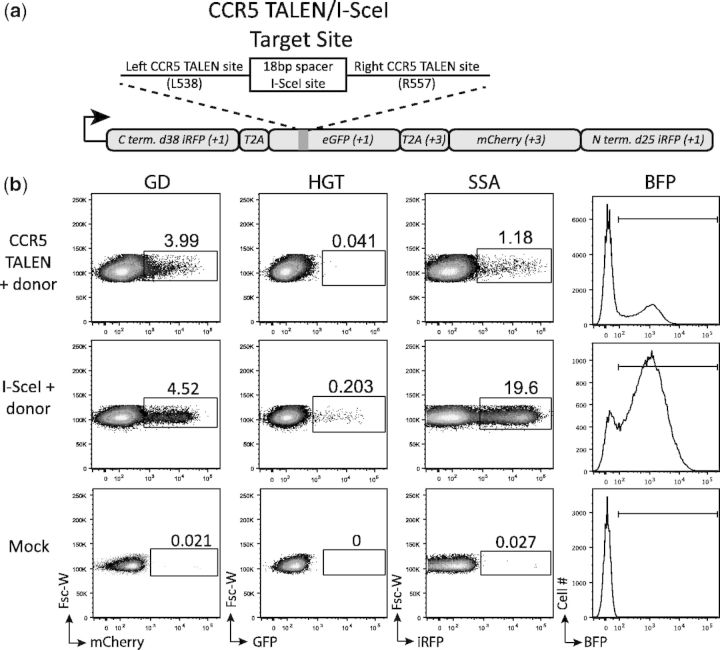
Influence of nuclease platform on DNA repair outcome. (**a**) Diagram of the SSA-TLR containing the CCR5 TALEN/I-SceI target site. Arrow represents promoter and initial *iRFP* start codon. Reading frames relative to the initial *iRFP* start codon are indicated in parentheses. (**b**) Flow cytometric analysis of HEK293T SSA-TLR with CCR5 TALEN (I-Sce I spacer) target site after transfection with TALEN or transduction with I-SceI and donor IDLV. BFP expression corresponds to nuclease transfection/transduction efficiency.

Because there are potentially important distinctions between designer endonuclease platforms, such as break polarity (HE leave 3′-overhangs, FokI-based enzymes leave 5′-overhangs) and propensity to end hold, we have been interested in exploring whether DSB breaks induced by nucleases from differing platforms are resolved in a similar fashion by cellular DNA repair machinery. In order to directly compare resolution by SSA, GT and GD for TALEN-induced versus HE-induced breaks, we designed an SSA-TLR containing a target site for a previously developed CCR5 TALEN (13,34), and incorporated an I-SceI target site within the 18-bp spacer between the two TALEN-binding sites (Figure 5a). Thus, breaks generated by the TALEN and I-SceI should fall within a few base pairs of each other within the spacer sequence. Following transduction/transfection of HEK293T cells containing this reporter with either I-SceI or the TALEN pair with and without donor template, we assessed SSA, GD and GT via flow cytometry (Figure 5b). While I-SceI-induced breaks resulted in frequencies of SSA, GD and GT in this cell line that were similar to those observed with the SSA-TLR containing the I-SceI target only, surprisingly, the TALEN-induced breaks in this same cell line were resolved with an extremely low frequency of GT, with a moderate frequency of SSA and GD (iRFP+ 1.8 ± 0.6, GFP+, 0.03 ± 0.02, mCherry+ 1.5 ± 0.2). We verified that this result was not an idiosyncratic property of this TALEN pair, as transfection of the same constructs into a TLR reporter with the identical target site led to easily detectable frequency of GT, (Supplementary Figure S7), suggesting that the observed differences in repair pathway utilization between I-SceI and the TALEN in the SSA-TLR are due to intrinsic properties of the nuclease reagent.

## DISCUSSION

While the current endonuclease-induced genome editing paradigm relies on the aleatory resolution of DNA breaks, the continued development of molecular strategies to bias repair towards a desired outcome is an important next step towards enabling rapid, precise and efficient site-specific genome modification. Towards this goal, we developed two novel fluorescent DNA repair reporters based on our previously characterized TLR that extend the high-throughput comparative analysis of DNA break resolution to new genome engineering contexts.

Chromatin status is known to regulate the accessibility of DNA-binding proteins to their targets (24,35,36); however, its effect on the efficiency and outcome of genome editing induced by DNA breaks has only recently begun to be explored (37–41). By driving the expression of a basic TLR with a viral promoter that is prone to silencing by CpG methylation (42,43), we were able to provide a direct comparative measure of GT and GD at a locus that has become transcriptionally silenced. In these experiments, we showed that local DNA methylation reduces the efficiency of genome editing induced by the endonuclease I-Sce I. Since the I-Sce I target site itself contains no CpG site, we expect that DNA methylation does not directly impair DNA binding and cleavage by steric hindrance, but rather through reduced accessibility to the target via chromatin compaction (44,45). Interestingly, we observed a more severe effect on GT as opposed to GD in repressed loci, consistent with the hypothesis that heterochromatin may limit extensive end resection (46). This work complements previous findings on the effect of donor DNA template chromatization (47) and suggests that inaccessible DNA may impede genome editing efforts. The AR-TLR could further be used to determine whether chromatin modifiers exclusively recruited to the chromosomal target site can influence the efficiency and outcome of DNA repair.

The SSA-TLR allowed us to efficiently and directly compare GT, GD and SSA at the same break-site. Our results using I-SceI indicate that an I-SceI-induced break frequently undergoes extensive 5′-end resection, such that if substantial (>500 bp) repetitive elements are proximal to the break-site, SSA is often used. This result is surprising in light of previous work that has shown quite low SSA rates in primary cells (20), even when using I-SceI-induced breaks. Thus, additional experimentation is needed to determine the generality of this observation, and using reporters with varying repeat homology lengths may be one approach to assess repair in different contexts. Nevertheless, as repetitive elements are interspersed widely throughout the human genome, the SSA-TLR offers a new model system for studying DNA repair pathway utilization when DNA breaks occur within areas of repetitive homologous sequences. Also surprising was the observation of markedly different repair pathway utilization following a TALEN-induced break versus an I-SceI-induced break: TALEN-induced breaks resulted in extremely inefficient SSA within a repetitive context, and nearly undetectable rates of GT. Whether the difference in repair pathway utilization between I-SceI versus TALEN-induced breaks is the result of the different polarity of the breaks, other aspects of the biochemistry of the two enzymes (e.g. end holding), or some other variable is unclear. However, the striking differences observed indicate that choice of nuclease platform can have an important influence on the outcome of a gene editing procedure, and emphasize the importance of continued comparative analyses involving multiple nuclease platforms.

## SUPPLEMENTARY DATA

Supplementary Data are available at NAR Online.

Supplementary Data
