# Supplementary material for: Novel fluorescent genome editing reporters for monitoring DNA repair pathway utilization at endonuclease-induced breaks
Source: Nucleic Acids Res. 2013 Oct 9;42(1):e4. doi: 10.1093/nar/gkt872 (PMC3874187; doi:10.1093/nar/gkt872)
Supplement: Supplementary Data [file supp_42_1_e4__index.html]

Novel fluorescent genome editing reporters for monitoring DNA repair pathway utilization at endonuclease-induced breaks — Novel fluorescent genome editing reporters for monitoring DNA repair pathway utilization at endonuclease-induced breaks — Supplementary Data 

# Novel fluorescent genome editing reporters for monitoring DNA repair pathway utilization at endonuclease-induced breaks

## Supplementary Data

files

**Files in this Data Supplement:**

- Supplementary Data - pdf file
