# Supplementary material for: Novel fluorescent genome editing reporters for monitoring DNA repair pathway utilization at endonuclease-induced breaks
Source: Nucleic Acids Res. 2013 Oct 9;42(1):e4. doi: 10.1093/nar/gkt872 (PMC3874187; doi:10.1093/nar/gkt872)
Supplement: Supplementary Data [file supp_gkt872_nar-01222-met-f-2013-File007.pdf]

Supplementary Figure 1
Plasmid Maps
1. Active/Repressed TLR

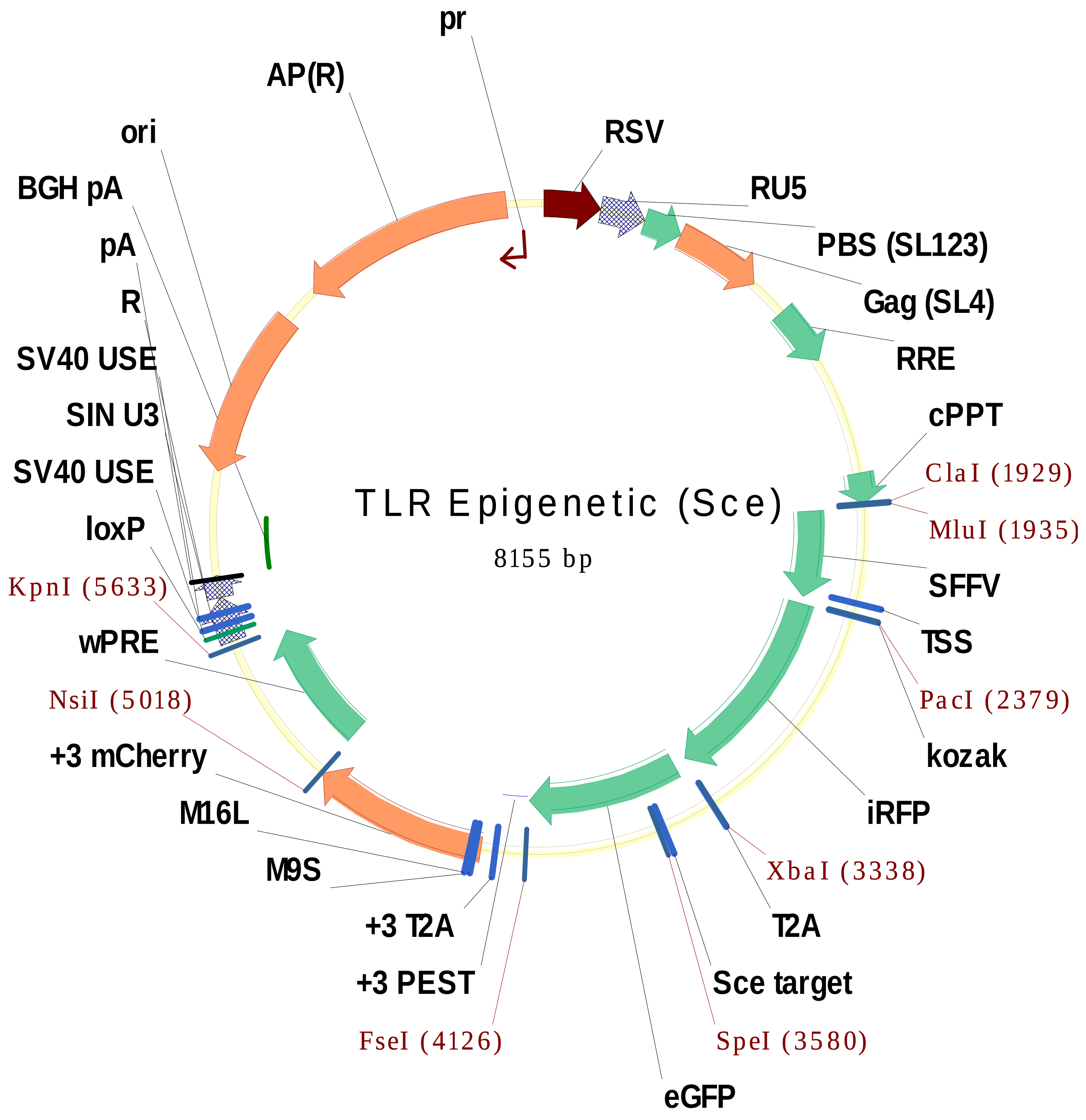

2. SSA-TLR

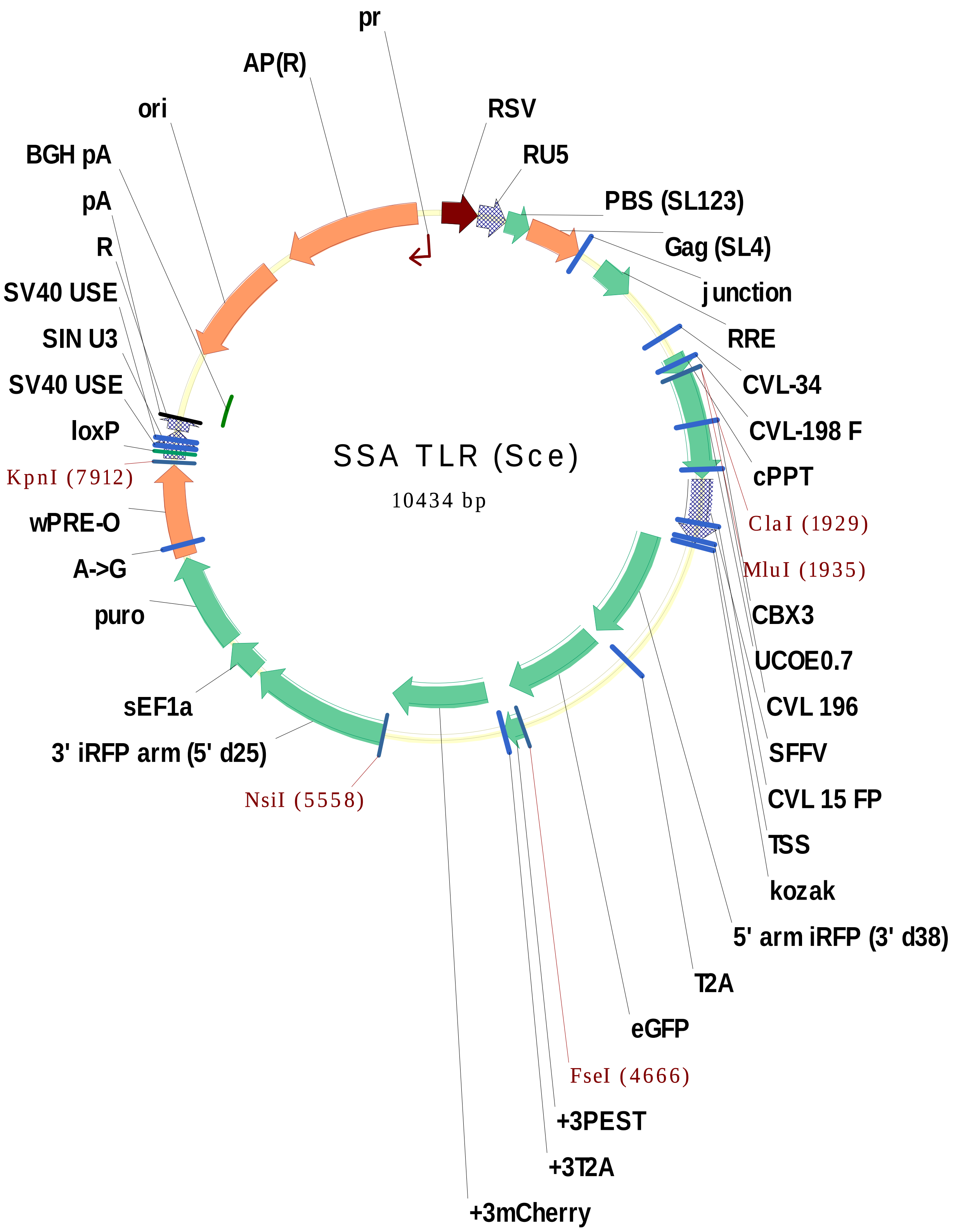
SSA TLR (Sce)
10434 bp
pr
AP(R)
ori
BGH pA
pA
R
SV40 USE
SIN U3
SV40 USE
loxP
KpnI (7912)
wPRE-O
A->G
puro
sEF1a
3' iRFP arm (5' d25)
NsiI (5558)
RSV
RU5
PBS (SL123)
Gag (SL4)
junction
RRE
CVL-34
CVL-198 F
cPPT
ClaI (1929)
MluI (1935)
CBX3
UCOE0.7
CVL 196
SFFV
CVL 15 FP
TSS
kozak
5' arm iRFP (3' d38)
T2A
eGFP
FseI (4666)
+3PEST
+3T2A
+3mCherry

3. pEX.d14GFP.sEF1a.Sce.BFP

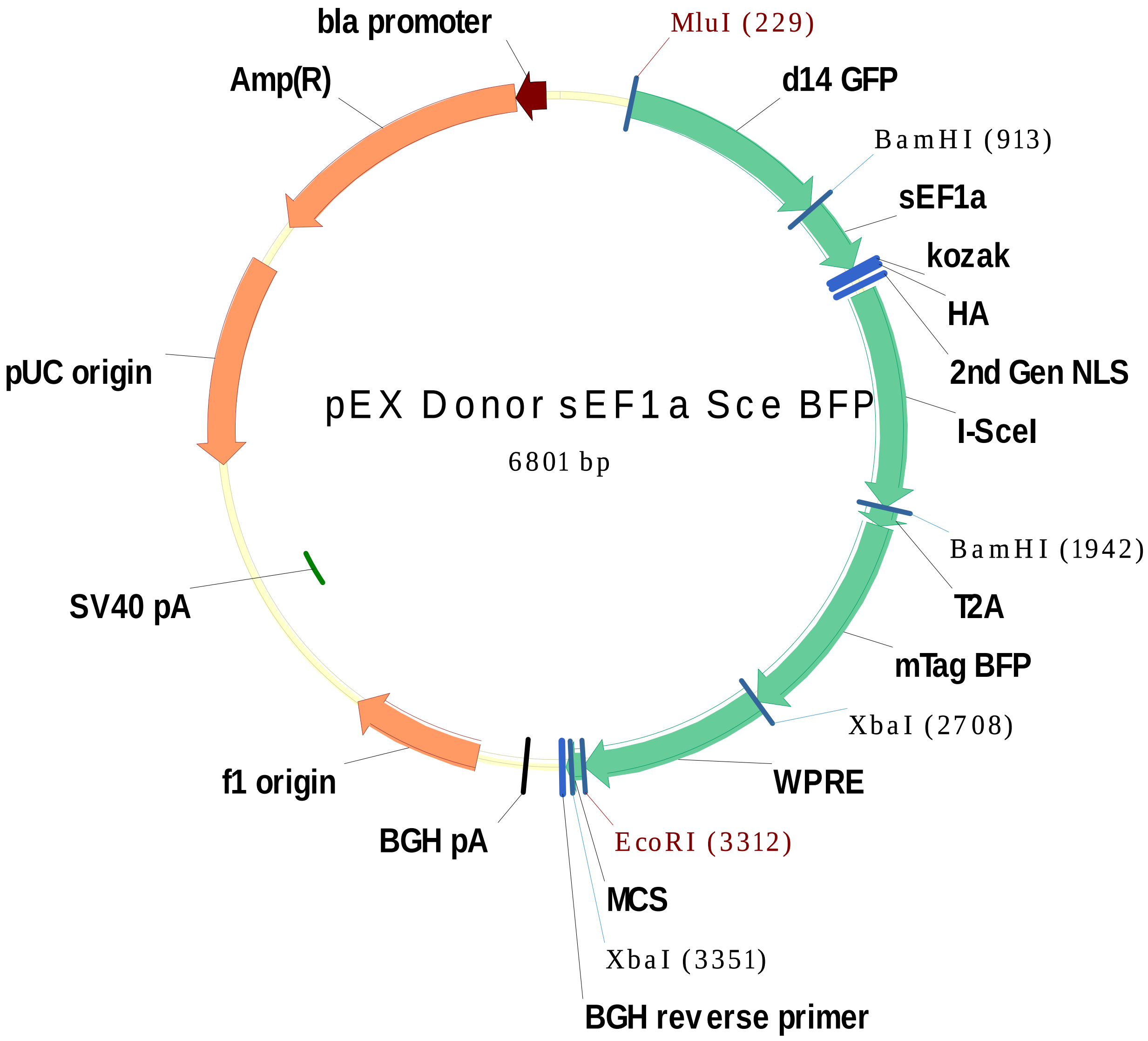

4. pCVL.SFFV.HA.NLS.I-Sce I.IRES.BFP

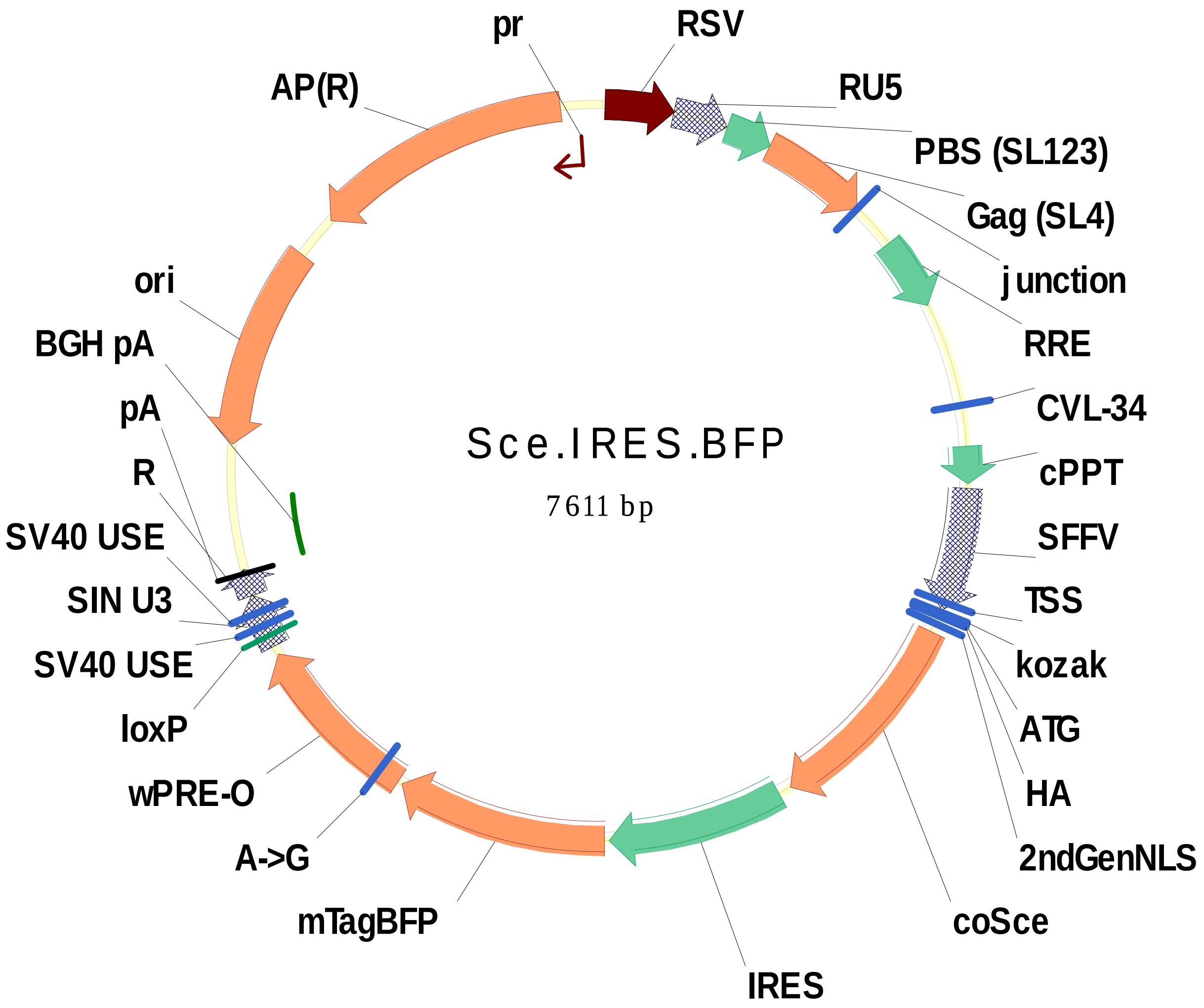

5. pCVL.SFFV.HA.NLS.I-Ani I Y2.IRES.BFP

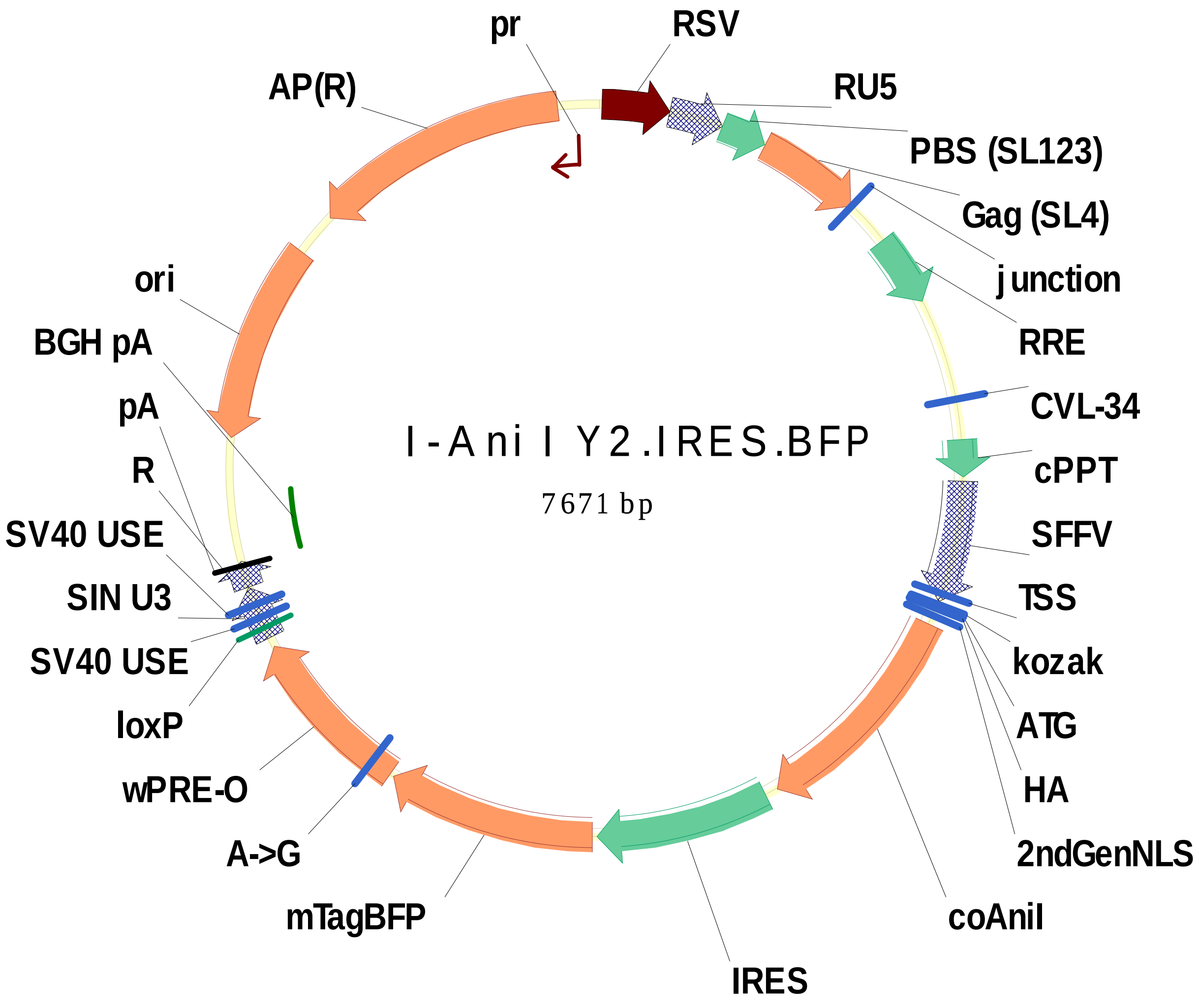

6. pCVL.SFFV.d14GFP

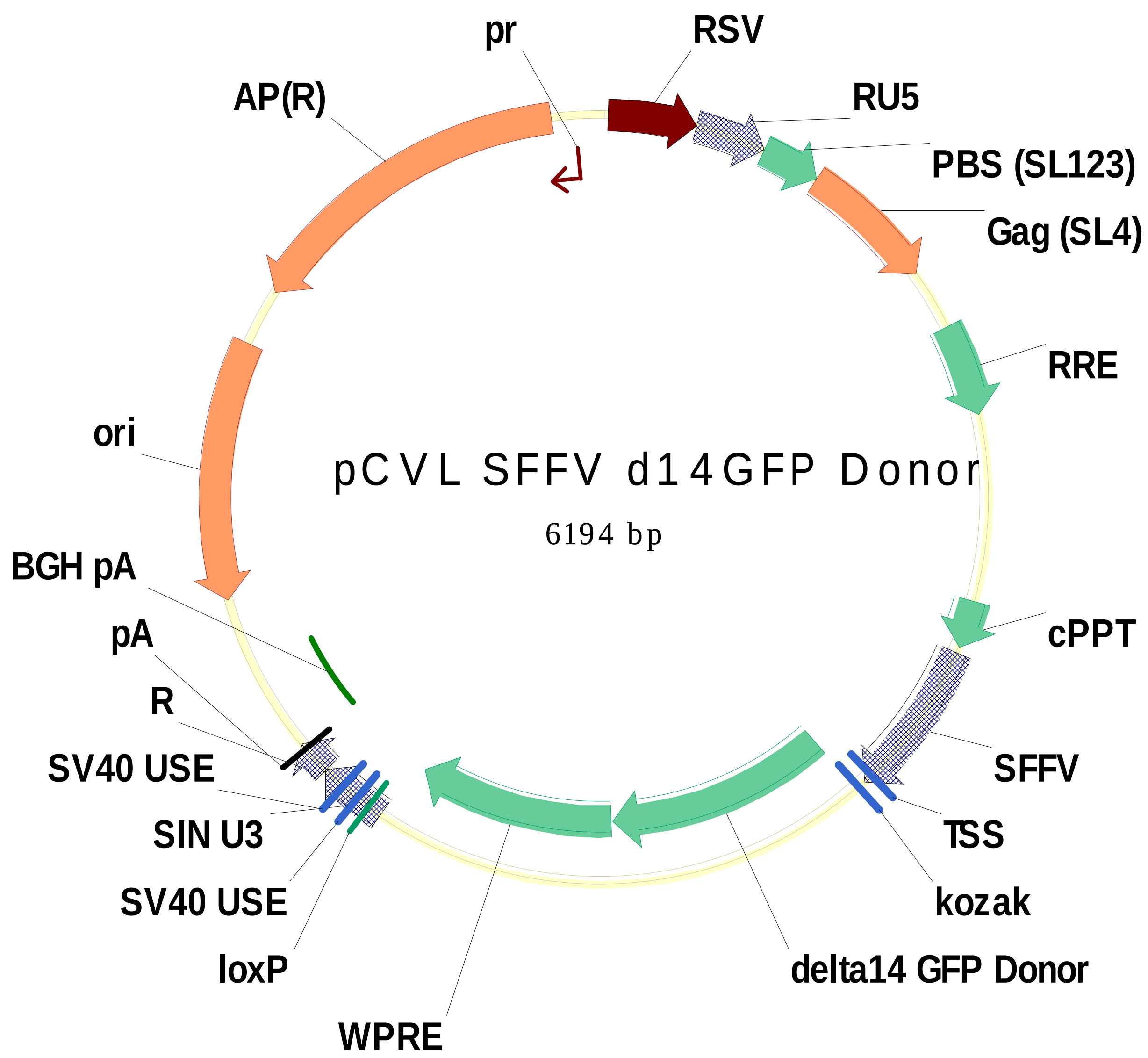

Supplementary Figure 2

a

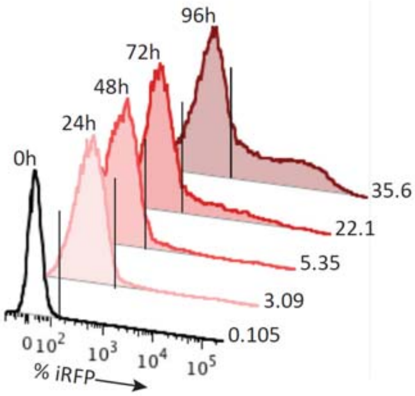

b

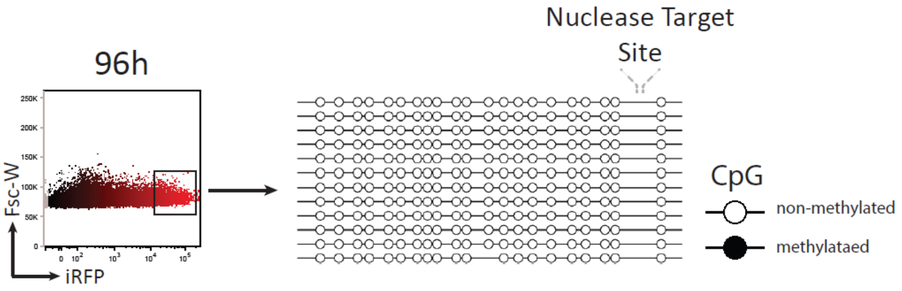

(a) Flow cytometric analysis depicting return of iRFP fluorescence in HEK293T AR-TLR iRFP- cells performed at 24 h intervals as cells were cultured in the presence of 5-aza-dC. Numbers inside plots indicate percentage of live cells.(b) Depiction of bisulfite sequencing results generated from the 5-aza-dC reactivated iRFP- cells isolated at 96 h of culture in 5-aza-dC. Representative sort gate shown in flow plot on left.

Supplementary Figure 3

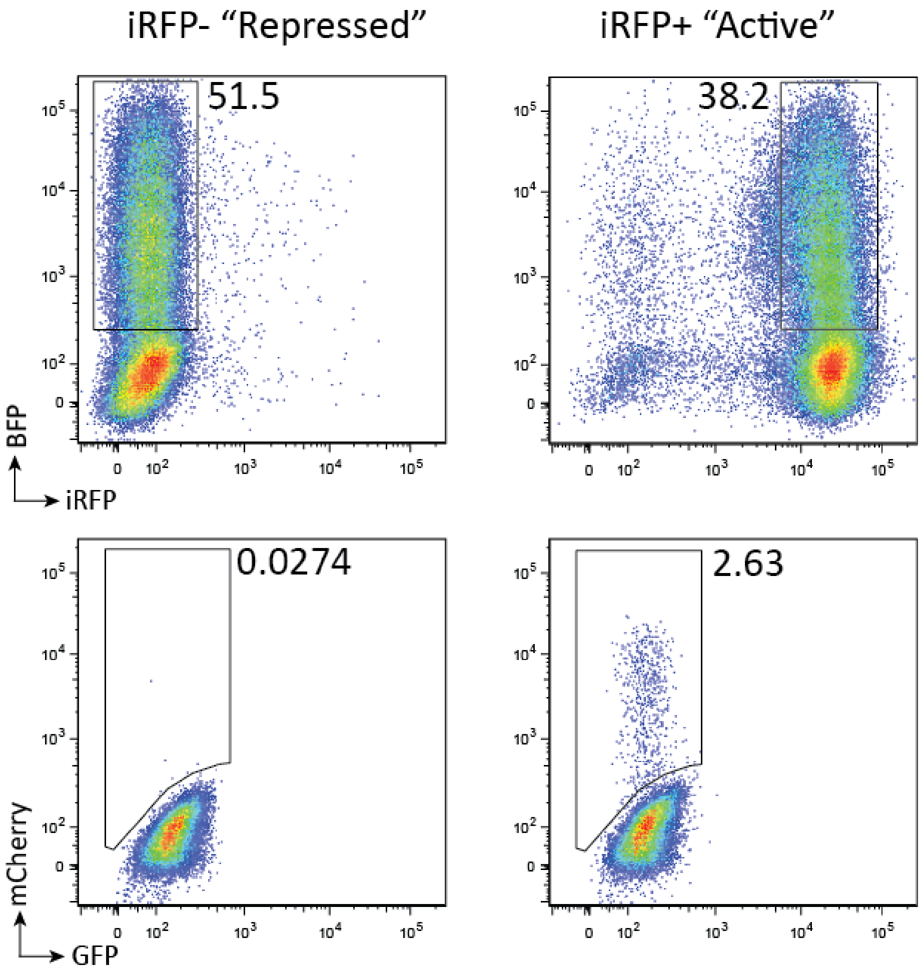

Representative flow cytometric readout showing fluorescent protein expression for Active/Repressed TLR cells that were used to assess overall mutagenesis rates via digest with recombinant I-Sce I.

Supplementary Figure 4

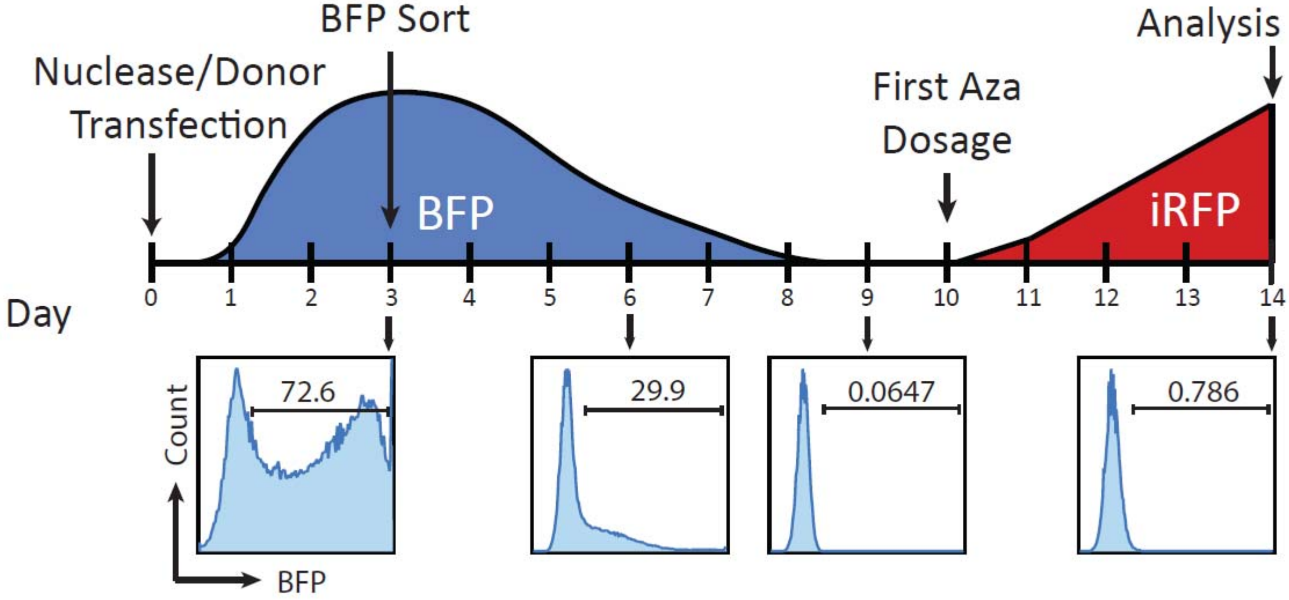

Schematic of experimental protocol for assessing HGT-mutagenic NHEJ ratios using the AR-TLR with histograms depicting corresponding levels of BFP (nuclease expression levels) fluorescence at specified time intervals.

Supplementary Figure 5

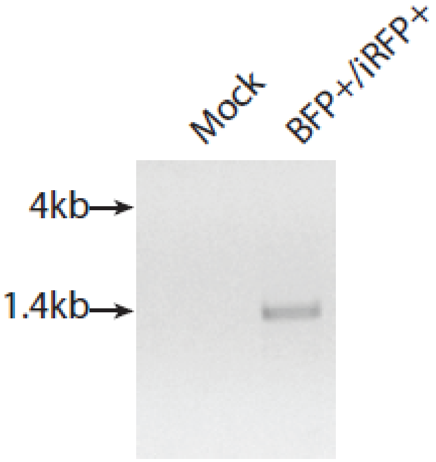

To confirm that the iRFP+ events indeed represented SSA, we looked for the characteristic loss of intervening sequence between the two arms in cells that had become iRFP+ after being treated with I-Sce I. This was accomplished by isolating genomic DNA from iRFP+ cells and generating PCR product from oligos placed just outside the iRFP arms. We observed a distinct 1.4KB band in the iRFP+ cells that corresponded to the predicted length of a reconstituted iRFP ORF following deletion of the intervening sequence.

Supplementary Figure 6

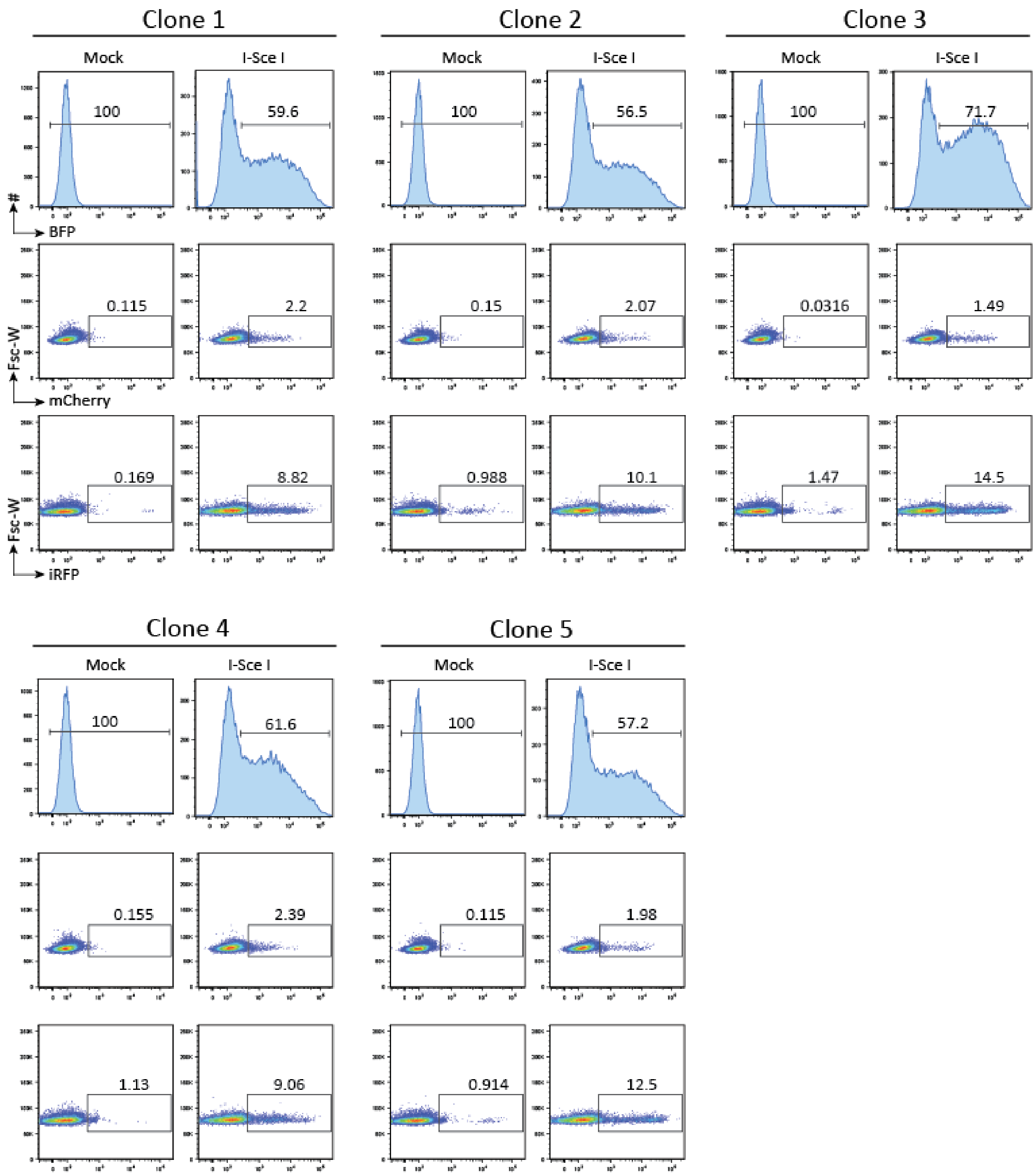

Flow cytometric readout of repair in five I-Sce I SSA-TLR cell line clones transiently transfected with I-Sce I expression plasmid and analyzed 72h post transfection. Based on this analysis clone 3 was selected for further experiments in this work. Mock corresponds to untreated. BFP fluorescence indicates expression of I-Sce I.

Supplementary Figure 7

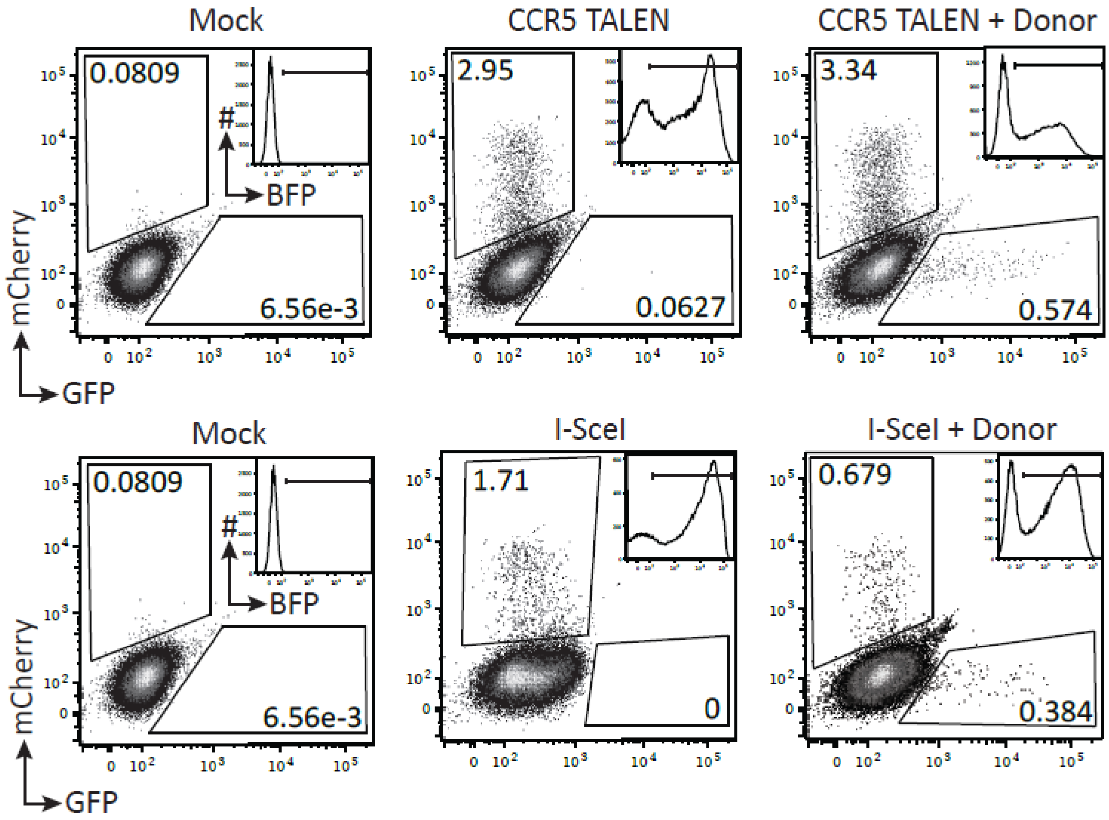

Flow cytometric readout of repair in Traffic Light Reporter 2.1 with CCR5 TALEN target site containing the I-Sce I target site as spacer. Cells were transfected with CCR5 TALEN pair or I-SceI with and without plasmid donor and analyzed 72h later. BFP expression corresponds to nuclease transfection efficiency.
